# Diagnosis and Treatment of Acute Pleural Effusion following Radioiodine Remnant Ablation Post Lobectomy for Thyroid Cancer

**DOI:** 10.3390/diagnostics12122982

**Published:** 2022-11-28

**Authors:** Xian Qiu, Pengwen Wang, Ri Sa, Lin Cheng, Yuchen Jin, Hongjun Song, Libo Chen

**Affiliations:** 1 Department of Nuclear Medicine, Shanghai Sixth People’s Hospital Affiliated to Shanghai Jiao Tong University School of Medicine, 600 Yishan Road, Shanghai 200233, China; 2 Department of Thyroid Surgery, Panshi Hospital, 1 Kangfu Road, Panshi 132300, China; 3 Department of Nuclear Medicine, The First Hospital of Jilin University, 71 Xinmin St., Changchun 130021, China

**Keywords:** differentiated thyroid cancer, radioiodine remnant ablation, ^131^I-lobectomy, pleural effusion, thoracic duct

## Abstract

Radioiodine remnant ablation (RRA) was previously demonstrated to be a safe and effective alternative to completion thyroidectomy for patients with differentiated thyroid cancer (DTC). However, its side effects have not been fully investigated, particularly in patients with lobectomy. We reported a young euthyroidal female who underwent RRA post lobectomy and lymph node dissection for papillary thyroid cancer, whose post-ablation ^131^I-whole-body scan accidentally showed diffuse radioiodine distribution on chest-mimicking pulmonary metastases. Immediately-added single-photon emission computed tomography/computed tomography (SPECT/CT), nevertheless, revealed a ^131^I-accumulating swollen left thyroid lobe and emerging pleural effusion, which relieved after short-term treatment with prednisone. In summary, acute pleural effusion ascribed to RRA-induced thoracic duct compression was reported for the first time. ^131^I-lobectomy-induced pleural effusion could be precisely diagnosed by SPECT/CT and efficiently manipulated via treating radiation thyroiditis with the short-term administration of corticosteroid.

## 1. Introduction

Thyroid cancer is among the most common malignancies, with a steadily and rapidly increasing global incidence. In 2020, there were approximately 586,000 new cases worldwide, ranking it in 9th place [1]. Differentiated thyroid cancer (DTC), including papillary thyroid cancer, follicular thyroid cancer, and oncolytic thyroid cancer, accounts for >90% of all thyroid cancers [2,3]. Lung represents the most commonly involved organ by metastases from DTC, followed by bone [4], whereas metastasis to pleural is much less common, occupying nearly 0.6% [5,6,7,8].

Thyroidectomy followed by ^131^I therapy and L-thyroxine therapy represents the mainstay procedure to manage DTC. Recently, owing to the optimistic prognosis of patients with DTC and the popularization of the latest American Thyroid Association (ATA) guidelines, lobectomy has been suggested as an initial surgical procedure in DTC patients with lower risk of persistent/recurrent or metastatic disease [9,10]. Although lobectomy could largely avoid surgery-related complications, completion thyroidectomy may still be needed in clinical settings of patients with multicentric disease, unexpected extrathyroidal extension, lymph node involvement, etc. [11].

Radioiodine remnant ablation (RRA), a form of oral ^131^I therapy, represents a conventional treatment modality for patients with DTC. Via eradicating the thyroid remnant, RRA is of great value to simplify response classification and facilitate dynamic risk stratification [12,13,14]. Moreover, accompanied post-ablation ^131^I whole-body scan (WBS) in combination with single-photon emission computed tomography/computed tomography (SPECT/CT) is of incremental value in disease surveillance and plays a vital role in the management of persistent/recurrent or metastatic disease [15]. At present, RRA has become a major part of therapeutic ^131^I administration, which also contains radioiodine adjuvant treatment for occult disease and radioiodine oncolytic treatment for known disease.

Recently, the ablation of the remaining whole-thyroid lobe by ^131^I, i.e., ^131^I-lobectomy, has been demonstrated to be a non-invasive, safe, and effective alternative to completion thyroidectomy for patients with DTC, which is especially favorable for those who are suffering from recurrent laryngeal nerve injury or parathyroid gland damage due to prior surgery [11,16]. However, the side effects of the ^131^I-lobectomy have not been sufficiently investigated, except for neck swelling and salivary disturbances [17,18,19].

Herein, we reported a case of ^131^I-lobectomy-induced acute pleural effusion mimicking lung metastatic DTC in a young female. Diagnostic procedures, treatment outcomes, and scientific hypothesis were described in detail.

## 2. Case Description

**Figure 1:**
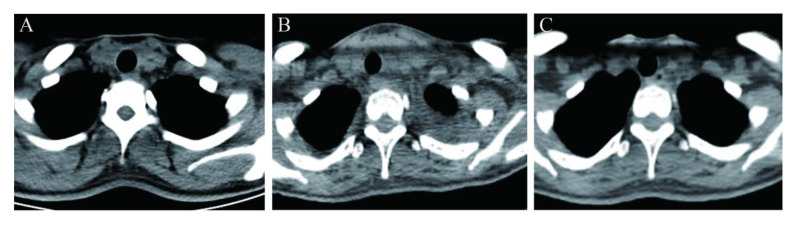
Neck CT images showing the remaining left thyroid lobe before ^131^I administration (**A**), and 3 days (**B**) and 9 days (**C**) post ^131^I administration.

**Figure 2:**
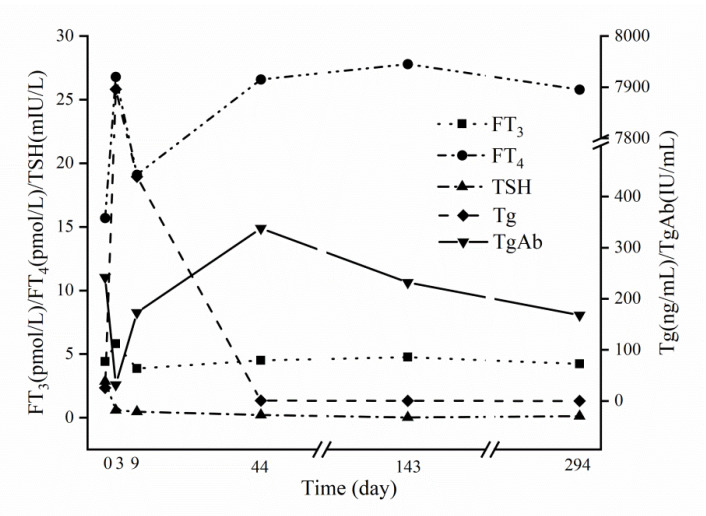
Dynamic changes in serum parameters from day 0 to day 294 after ^131^I administration. FT_3_, free triiodothyronine (normal range: 3.67–6.00 pmol/L); FT_4_, free thyroxine (normal range: 7.50–21.10 pmol/L); TSH, thyroid-stimulating hormone (normal range: 0.34–5.60 mIU/L); Tg, thyroglobulin (normal range: 3.50–77.00 ng/mL); and TgAb, anti-Tg antibody (normal range: 0.00–115.00 IU/mL).

A 29-year-old female, who had undergone lobectomy plus therapeutic lymph node dissection for a 1.2-cm papillary thyroid cancer nodule with multiple nodal metastases, was referred to our institution for RRA, when she orally took L-thyroxine at a dose of 62.5 milligram (mg) per day, with a body weight of 55 kilogram (kg). After four weeks of L-thyroxine withdrawal before RRA, plain computed tomography scan showed no abnormal findings in the chest (no evidence of pleural disease), and the remaining left thyroid lobe (Figure 1A) measured 13 millimeter (mm) × 12 mm × 41 mm with normal blood flow by ultrasonography. All laboratory workup showed normal blood cell count, hepatic function, and thyroid function, with a thyroid-stimulating hormone (TSH) level of 2.86 mIU/L, a free triiodothyronine (FT_3_) level of 3.92 pmol/L, and a free thyroxine (FT_4_) level of 15.70 pmol/L (Figure 2).

**Figure 3:**
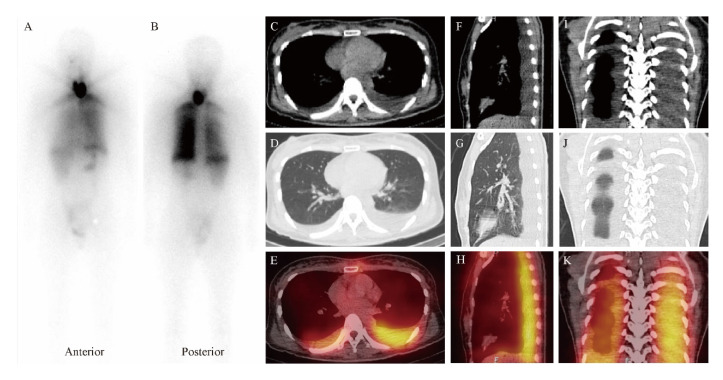
Whole-body scan ((**A**), anterior view; (**B**), posterior view) on day 3 post ^131^I administration, showing ^131^I distribution in left thyroid lobe and chest. SPECT/CT images of chest ((**C**–**E**), transaxial; (**F**–**H**), sagittal; and (**I**–**K**), coronal) showing bilateral pleural effusion, predominantly in the left side. Neither abnormal uptake nor lesion was found in either lung.

An activity of 5.55 GBq (150 mCi) of ^131^I was then orally administered for lobe ablation. Simultaneously, prednisone at a dose of 20 mg three times daily was initiated and sustained for a week. On day three post ^131^I administration, WBS showed ^131^I distribution in the neck and chest (Figure 3). To precisely localize the ^131^I accumulation sites and reveal the underlying mechanism, SPECT/CT imaging was immediately added, showing a severely swollen left thyroid lobe (Figure 1B) and bilateral pleural effusion without solid metastatic lesions (Figure 3). Although the patient felt mild chest tightness at that time, respiratory symptoms and signs were not observed. Laboratory examinations indicated a leukocyte count of 10.8 × 10^9^/L with an increase in the proportion of neutrophils, a TSH level of 0.59 mIU/L, an FT_3_ level of 5.80 pmol/L, and an FT_4_ level of 26.80 pmol/L (Figure 2).

**Figure 4:**
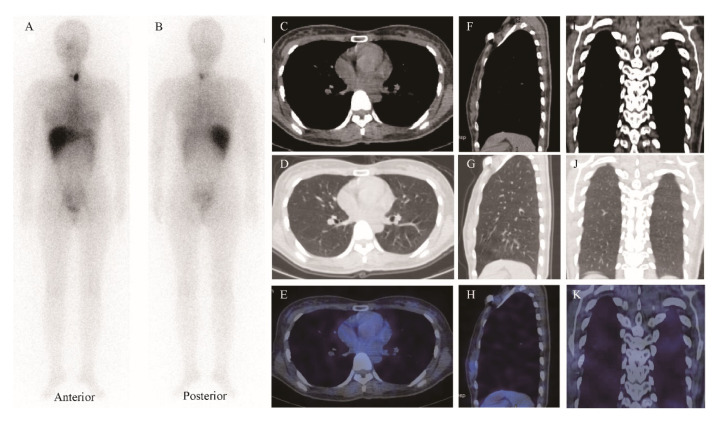
Whole-body scan ((**A**), anterior view; (**B**), posterior view) on day 9 post ^131^I administration, showing ^131^I accumulation only in the thyroid lobe remnant. SPECT/CT images of chest (**C**–**E**), transaxial; (**F**–**H**), sagittal; and (**I**–**K**), coronal), showing neither ^131^I accumulation in the chest nor pleural effusion.

A diagnostic aspiration was refused by the patient, due to her experiencing no other discomfort. On day nine after ^131^I administration, when her chest tightness had disappeared, a follow-up WBS and SPECT/CT revealed no ^131^I accumulation in the chest, and the pleural effusion was absorbed completely (Figure 4). CT showed that the ^131^I-induced thyroiditis relieved robustly with a resetting trachea (Figure 1C). Moreover, the TSH level of 0.47 mIU/L, the FT_3_ level of 3.86 pmol/L, the FT_4_ level of 19.10 pmol/L, the blood cell count, and the serum albumin level were all within normal ranges (Table 1). L-thyroxine replacement therapy at a dose of nearly 2 mg/kg of body weight was started two weeks after ^131^I administration, yielding a favorable TSH level of 0.21 mIU/L, an FT_3_ level of 4.51 pmol/L, and an FT_4_ level of 26.60 pmol/L, after L-thyroxine replacement for one month.

Ten months after the ^131^I-lobectomy, the successful RRA was verified and the excellent response was achieved in this patient, based on the outcomes of serum test and ultrasonography examination [14,18]. Specifically, the TSH level of 0.02 mIU/L, the thyroglobulin (Tg) level of 0.12 ng/mL, and the anti-Tg antibody (TgAb) level of 168.00 IU/mL were documented on day 294 days post RRA (Figure 2). Moreover, ultrasonography showed that the left thyroid gland gradually shrank to 15 mm × 18 mm × 35 mm, 13 mm × 17 mm × 25 mm, and 9 mm × 12 mm × 25 mm at one, four, and ten months post RRA, respectively. Meanwhile, neither blood flow in the ablated thyroid lobe nor nodal disease in the neck was found during the above ultrasound examinations.

## 3. Discussion

An increase in thyroid lobectomy for patients with DTC has become a trend in the latest decade, especially after the 2015 ATA guidelines were issued [9,11]. Consequently, more challenges in RRA may be met during real-word nuclear medicine practice in treating DTC patients who have undergone thyroid lobectomy. Our patient was classified as intermediate risk, mainly based on her postoperative pathological findings, and ^131^I therapy should be considered, according to the 2015 ATA guidelines [10]. Compared with completion thyroidectomy, which might carry complications for patients, a non-invasive and safe RRA may be chosen by patients [11] because RRA is critical to facilitate response classification post initial treatment and dynamic recurrence risk stratification by Tg measurement and WBS. Consistent with other studies [14,20,21,22], the successful RRA and excellent therapeutic response allowed our patient a decrease in the frequency of follow-up and the degree of TSH suppression because a recurrence risk of only 1–4% and a disease-specific death of merely < 1% could be expected [10]. Moreover, for the first time, acute pleural effusion was reported as a new side effect of RRA, which was precisely diagnosed by SPECT/CT fusion imaging and efficiently treated by the short-term use of corticosteroid.

As is well known, DTC metastases were the most common pathologically malignant etiologies of ^131^I uptake in the chest views of planar imaging, including spot view and WBS. Owing to the improvement of diagnostic accuracy by incorporating hybrid SPECT/CT in the last two decades, most causes of ^131^I uptake in rare clinical settings could be identified, with incremental value in the management of patients with DTC, as previously described by our group [23,24]. Recently, extremely scarce cases of solitary breast metastasis from DTC and transplantation in endoscopic thyroidectomy were reported [25,26]. Furthermore, ^131^I-avid malignancies beyond DTC have been identified by ^131^I WBS with or without SPCET/CT, such as primary lung cancer, gastric adenocarcinoma, metastatic salivary gland tumor, and papillary meningioma [27].

Pathologically benign etiologies in the chest have also been recognized to accumulate ^131^I, including bronchiectasis, respiratory bronchiolitis, pulmonary tuberculosis, pulmonary aspergilloma, breast fibroadenoma, pleuropericardial cyst, hyperplastic thymus, bronchial atresia with mucocele, and pulmonary sequestration [27,28,29]. Potential mechanisms of ^131^I uptake in these entities were deemed as increased concentration of ^131^I due to the hyperemia of the inflamed mucosa, the leakage of ^131^I into bronchial tree or lung parenchyma because of increased permeability, and the accumulation of tracheobronchial secretions due to decreased clearance [30]. In contrast to DTC lesions, which commonly show persistent ^131^I uptake, chronic pulmonary inflammation usually manifest transient ^131^I uptake, which may be revealed by repeated WBS. More importantly, significant information simultaneously provided by the diagnostic CT compartment of SPECT/CT plays a vital role in the differential diagnoses.

Additionally, ^131^I distribution viewed in the chest has even been found in physiological conditions. Firstly, esophageal retention could be misdiagnosed, when a planar image illustrates focal or diffuse uptake rather than linear uptake. The underlying mechanisms involve the retention of saliva due to decreased esophageal motility, mechanical obstruction, or pooling of saliva in the posterior pharyngeal pouch, secondary to achalasia, esophageal stricture, and Zenker’s diverticulum [31,32,33]. Although delayed planar images may be helpful for the final diagnosis, since the retention of ^131^I in the esophagus changes or disappears with time, an immediately added SPECT/CT may help reveal the cause readily, which is similarly applied to identify physiological ^131^I uptake by gastric and colonic mucosa of aberrant locations in the chest [34]. Secondly, lactating breast can take up ^131^I, due to the active expression of the Na/I transporter, which may be symmetrical, asymmetrical, or unilateral, mimicking lung metastases [35]. The diagnosis might be explicit by integrating the patient’s expression of galactorrhea and/or elevated prolactin levels [36]. Thirdly, ^131^I uptake in ectopic thyroid tissue viewed in the chest is traditionally considered as a false-positive finding in the situation of the diagnosis of thyroid cancer, which could have been easily misdiagnosed as DTC metastasis due to the difficulties in the differential diagnosis in WBS. However, combined with SPECT/CT, a commonly midline-location is helpful to establish the diagnosis of ectopic thyroid tissue [34,37].

Even more than the above, external contamination with body secretions may mimic lung or bone lesions, owing to ^131^I distribution in the chest on the planar scan. ^131^I-containinig salivary, nasopharyngeal, trachea-bronchial secretions, or sweat can contaminate the skin and/or garment by coughing, sneezing, perspiring, and tobacco chewing and can be recognized with the aid of SPECT/CT, by comparing planar images before and after clearance, and by changing wearing [38,39,40].

**Figure 5:**
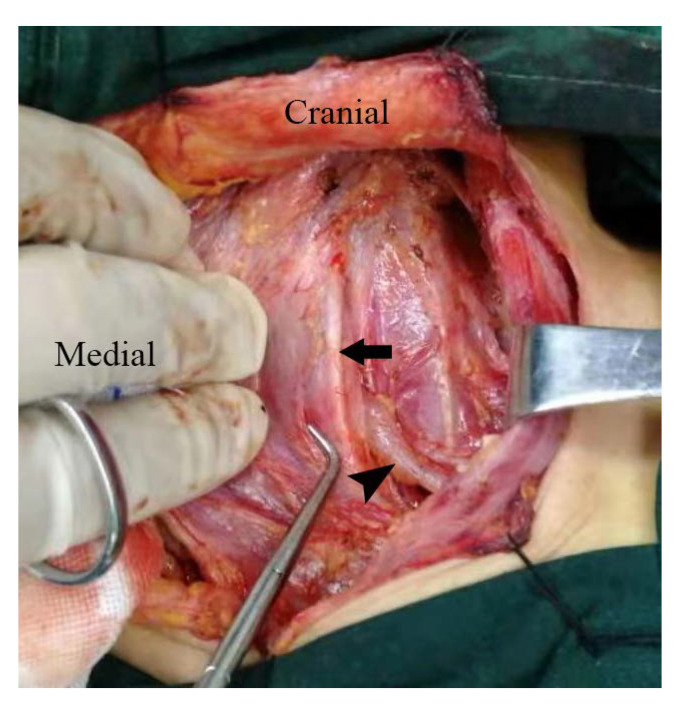
Surgical anatomy of neck illustrating carotid sheath (arrow) and thoracic duct (arrow head) of another patient, showing that thoracic duct passes posterior to the left common carotid artery and injects into the left venous angle.

As shown in Figure 3, WBS combined with SPECT/CT revealed ^131^I accumulation in the chest, owing to the prominent pleural effusion. To the best of our knowledge, tiny pleural effusion has been once suspected as a false-positive finding by WBS and SPECT/CT in a case report [41]. Mechanistically, an increased pleural effusion reflects a disturbance of equilibrium between the production and resorption of fluid in the pleural cavity, which is usually caused by primary and secondary cancers, hypothyroidism, congestive heart failure, liver disease, and so on [42,43,44]. Nevertheless, our patient had neither the medical history mentioned above nor relevant abnormal laboratory findings. Notably, the swollen left thyroid lobe compressing the surrounding structures and bilateral pleural effusion with the left predominant relieved in parallel, indicating compression of the thoracic duct as a potential mechanism according to anatomy (Figure 5) [45]. We, therefore, postulated that the thoracic duct was compressed by a swollen thyroid, causing acute and massive lymphatic fluid to exude in the pleural cavity.

Traditionally, L-thyroxine replacement therapy was usually started 48 h after ^131^I administration in patients with DTC who had undergone total or near-total thyroidectomy. However, the optimal timing to start L-thyroxine replacement therapy after ^131^I administration remains unclear in DTC patients post lobectomy, due to the lack of pertinent data. Based on the dynamic data on serum parameters we continuously obtained (Figure 2), the FT_4_ level dropped promptly after a transient enhancement on day nine, and L-thyroxine replacement therapy was started at two weeks after ^131^I administration, yielding favorable values of TSH, FT_3,_ and FT_4_. Thus, we deem that two weeks after ^131^I administration represents an appropriate timing to start L-thyroxine replacement therapy in this entity.

Notably, according to the ultrasound features of our patient during 10 months of follow-up after RRA, the remaining thyroid gland continuously shrank with the absence of blood flow, indicating a mummified thyroid lobe in line with the findings previously reported by our team [14]. Furthermore, the value of TgAb decreased gradually after a transient increase post the RRA (Figure 2), similar to the mode of change in the anti-TSH antibody level post RRA in patients with Graves’ disease [46]. It has been highlighted that TgAb should be detected together with Tg during the follow-up of patients with DTC due to its potential interference with Tg assay [47]. To date, nevertheless, the cut-off value of TgAb has not been established, to avoid interference [48,49]. In our patient, however, the declining Tg level accompanied by the decreasing TgAb level indicated a favorable outcome, i.e., an excellent response to the initial management. It was reported by other studies that patients with decreased TgAb levels could be associated with lower recurrence rate compared to those with an increased TgAb level during follow-up [50,51].

Acute side effects of RRA should not be ignored since they often bring patients divergent discomforts. Gastrointestinal discomforts represent the most common ^131^I therapy-related symptoms, in which nausea and vomiting usually occur within 36 hours after ^131^I administration. Salivary gland swelling, pain, and dysfunction, which may develop into dry mouth after patients are discharged from the hospital, have attracted continuous attention since there is no effective precaution approach [52]. Additionally, neck pain and swelling due to thyroiditis have been reported in up to 50–66% patients with lobectomy undergoing RRA, and such symptoms usually resolve after oral administration of paracetamol or corticosteroid for a few days [11]. It was reported that 8.3–26.5% of subjects who experienced moderate or severe symptoms recovered after taking prednisone 20–40 mg/day for 3 days, subsequently tapered over 7–10 days [20,53]. Instead of initiating prednisone when patients developed significant neck pain and swelling, nevertheless, our patients were prophylactically given corticosteroid in a short course. Moreover, the dose of 60 mg daily in our case was higher than those reported in the previous studies [20]. All of the modulations above might be attributable to the more efficient control of ^131^I-induced thyroiditis in the intact lobe. As expected, our patient did not suffer from neck pain and dyspnea, except for neck swelling and mild chest tightness. Notably, expect for the transient and mild increase in leukocyte count, there were no other adverse events caused by the short-term use of corticosteroid, indicating an excellent safety profile of this medication regimen [54].

## 4. Conclusions

Our case report indicated that the ^131^I-lobectomy represents an acceptable alternative to completion thyroidectomy in patients with DTC. We firstly identified acute pleural effusion attributable to RRA-induced thoracic duct compression, which could be precisely diagnosed by SPECT/CT and efficiently manipulated by treating radiation thyroiditis with the short-term use of corticosteroid.

## Figures and Tables

**Table 1:** Laboratory data before and after ^131^I administration.

	Before ^131^I Administration	Day 9 after ^131^I Administration	Day 16 after ^131^I Administration
Leukocyte (×10^9^/L)	4.2	4.9	5.3
Neutrophil (×10^9^/L)	2.4	4.1	4.0
Albumin (g/L)	55	46.5	50.0
ALT (U/L)	18	14	12
AST (U/L)	20	15	16
TBIL (umol/L)	12.7	8.2	7.4
DBIL (umol/L)	2.5	2.0	1.8
Creatinine (umol/L)	54	49.5	55.4
eGFR-EPI (mL/min/1.73 m)	122.89	126.46	121.86
proBNP (ng/mL)	NA	34.21	NA

Leukocyte, (normal range: 3.5–9.5 × 10^9^/L); neutrophil, (normal range: 1.8–6.3 × 10^9^/L); albumin, (normal range: 35–55 g/L); ALT, alaninetransaminase (normal range: 0–65 U/L); AST, aspartate aminotransferase (normal range: 8–37 U/L); TBIL, total bilirubin (normal range: 0.0–18.0 umol/L); DBIL, direct bilirubin (normal range: 0.0–6.0 umol/L); creatinine (normal range: 53–115 umol/L); and proBNP, pro B type natriuretic peptide (normal range: 5.00–125.00 ng/mL). NA, not available.

## Data Availability

Not applicable.

## Abbreviations

DTC, differentiated thyroid cancer; ATA, American Thyroid Association; RRA, radioiodine remnant ablation; WBS, whole-body scan; SPECT/CT, single-photon emission computed tomography/computed tomography; TSH, thyroid-stimulating hormone; FT3, free triiodothyronine; FT4, free thyroxine; Tg, thyroglobulin; and TgAb, anti-Tg antibody.
